# Synthetic extracellular volume fraction without hematocrit sampling for hepatic applications

**DOI:** 10.1007/s00261-021-03140-6

**Published:** 2021-06-10

**Authors:** Narine Mesropyan, Patrick Kupczyk, Alexander Isaak, Christoph Endler, Anton Faron, Leona Dold, Alois M. Sprinkart, Claus C. Pieper, Daniel Kuetting, Ulrike Attenberger, Julian A. Luetkens

**Affiliations:** 1 Department of Diagnostic and Interventional Radiology, University Hospital Bonn, Venusberg-Campus 1, 53127 Bonn, Germany; 2 Quantitative Imaging Lab (QILab), Venusberg-Campus 1, 53127 Bonn, Germany; 3 Department of Internal Medicine I, University Hospital Bonn, Venusberg-Campus 1, 53127 Bonn, Germany

**Keywords:** Magnetic resonance imaging, Extracellular volume fraction, Liver fibrosis, Hematocrit

## Abstract

**Purpose:**

Calculation of extracellular volume fraction (ECV) currently receives increasing interest as a potential biomarker for non-invasive assessment of liver fibrosis. ECV calculation requires hematocrit (Hct) sampling, which might be difficult to obtain in a high-throughput radiology department. The aim of this study was to generate synthetic ECV for hepatic applications without the need for Hct sampling.

**Methods:**

In this prospective study participants underwent liver MRI. T1 mapping was performed before and after contrast administration. Blood Hct was obtained prior to MRI. We hypothesized that the relationship between Hct and longitudinal relaxation rate of blood (R1 = 1/T1_blood_) could be calibrated and used to generate the equation for synthetic Htc and ECV calculation. Conventional and synthetic ECV were calculated. Pearson correlation, linear regression and Bland–Altman method were used for statistical analysis.

**Results:**

180 consecutive patients were divided into derivation (*n* = 90) and validation (*n* = 90) cohorts. In the derivation cohort, native R1_blood_ and Hct showed a linear relationship (Hct_MOLLI_ = 98.04 × (1/T1_blood_) − 33.17, *R*^2^ = 0.75, *P* < 0.001), which was used to calculate synthetic ECV in the validation and whole study cohorts. Synthetic and conventional ECV showed significant correlations in the derivation, validation and in the whole study cohorts (*r* = 0.99, 0.97 and 0.99, respectively, *P* < 0.001, respectively) with minimal bias according to the Bland–Altman analysis.

**Conclusion:**

Synthetic ECV seems to offer an alternative method for non-invasive quantification of the hepatic ECV. It may potentially overcome an important barrier to clinical implementation of ECV and thus, enable broader use of hepatic ECV in routine clinical practice.

## Introduction

Chronic liver disease is a global public health concern and accounts for approximately 2 million deaths per year worldwide [1]. Liver cirrhosis as a consequence of chronic liver disease is currently the 11th most common cause of death globally and within the top 20 causes of disability-adjusted life years and years of life lost [2]. The detection and staging of liver fibrosis is of great clinical importance for treatment decisions and prognosis estimation, therefore, reliable tools are necessary in these patients. Although considered the gold standard, liver biopsy has its clear drawbacks and, therefore, is no longer routinely performed for staging and monitoring of liver fibrosis. Consequently, non-invasive techniques such as transient elastography and magnetic resonance elastography (MR-elastography) are increasingly preferred in order to diagnose and grade liver fibrosis. Especially MR-elastography is considered one of the most accurate non-invasive technique for liver fibrosis assessment with accuracies varying from 89 to 95% depending on fibrosis stage and underlying liver disease [3, 4]. However, it can be associated with a high technical failure rate, i.e., in patients with massive ascites, obesity or iron deposition [5, 6].

In differentiating between normal and diseased liver parenchyma, the concept of evaluating the T1 relaxation times was first mentioned in the 1980s. Hepatic fibrosis increases the T1 relaxation time of liver parenchyma due to an increase of extracellular matrix and protein concentration. T1 mapping techniques also allow the estimation of extracellular volume fraction (ECV) from native and post-contrast T1. ECV values are calculated from the change in relaxation rate (R1 = 1/T1) of blood and parenchyma corrected for the hematocrit (Hct) [7]. Therefore, calculation of ECV requires Hct sampling. MRI-derived ECV using T1 mapping techniques is currently of increased interest as a new non-invasive tool for liver fibrosis assessment [7–12]. There are already studies, demonstrating a high diagnostic performance of ECV in liver fibrosis assessment in both, animal and human models [7, 9]. ECV correlates with histological markers of liver fibrosis and has a high diagnostic performance for liver fibrosis assessment with accuracies up to 85% depending on underlying liver disease and fibrosis stage [7, 9, 13–15]. Furthermore, the longitudinal reflexivity (R1 = 1/T1) of blood is known to be in a linear relationship with blood Hct. It is determined by the water fractions of plasma and the erythrocyte cytoplasm, which undergo fast water exchange [16–21]. Previous cardiac MRI studies showed that ECV quantification without blood sampling, assuming a linear relationship between blood Hct and longitudinal T1 relaxation times (1/T1_blood_), is feasible [22, 23]. But there are still no studies showing whether it is also applicable for calculation of hepatic ECV. A synthetic ECV calculation would be beneficial considering the fact that liver fibrosis assessment and staging using T1 mapping techniques could be performed non-invasively and time-efficient directly after the MRI examination.

The hypothesis of our study was that a linear relationship between blood Hct and longitudinal T1 relaxation times (1/T1_blood_) could be used for synthetic Hct estimation, which permits synthetic ECV calculation without Hct sampling. The aim of this study was (1) to create a synthetic Hct regression model and (2) to investigate whether synthetic Hct can be used for reliable and valid calculation of synthetic ECV compared to conventional ECV.

## Materials and methods

This study was approved by the institutional review board. Written informed consent was obtained from all participants prior to MRI examination. From March 2019 to November 2020, consecutive patients with clinical indications for liver MRI examination were included in this study. Patients with and without chronic liver disease were included. Diagnosis of chronic liver disease was based on past medical history (including liver biopsy, clinical and laboratory examinations) and MRI (including MR-elastography). When necessary, the presence of significant fibrosis at MRI was assessed by MR-elastography as a reference standard using previous published cutoffs [3, 4]. Exclusion criteria were contraindications for contrast-enhanced MRI. Hematocrit samples were derived directly prior to MRI examination. According to the underlying liver disease, all patients were randomly split into the derivation and validation cohort. Clinical data and additional laboratory markers were recorded from the patient charts. Biochemical blood analyses were performed using standard tests and non-invasive scoring systems based on laboratory tests for assessment of liver fibrosis (aspartate aminotransferase-to-platelet ratio index (ARPI), fibrosis index based on the 4 factor (FIB-4), MELD score (Model of End Stage Liver Disease) and aspartate aminotransferase and alanine aminotransferase ratio (AST/ALT ratio (de-Ritis)) were calculated [24–26].

### Magnetic resonance imaging

All participants underwent MRI examination on a clinical whole-body 1.5-T system (Ingenia, Philips Healthcare) equipped with 32-channel abdominal coil with digital interface for signal reception. In addition to morphological sequences, patients underwent hepatic T1 mapping with a heart rate independent 10-(2)-7-(2)-5-(2)-3-(2) modified Look-Locker inversion recovery (MOLLI) acquisition scheme with internal triggering [27]. Technical parameters were as follows: time of repetition/time of echo 1.92/0.84 ms, flip angle 20°, parallel imaging factor 2, acquired voxel size 1.98 × 2.45 × 10 mm, reconstructed voxel size 1.13 × 1.13 × 10 mm, scan duration/breath-hold 14.0 s. For the post-contrast T1 maps, the same technique was used after 10 min of contrast agent application in the same positions as pre-contrast examinations. T1 maps were acquired in end-expiration [28]. For contrast-enhanced T1 mapping, a gadolinium-based contrast agent (Gadobutrol, 1.0 mmol/ml solution with 0.1 mmol per kilogram of body weight, Gadovist, Bayer Healthcare Pharmaceuticals) was administered as a single bolus with an injection rate of 1.5 ml/s. Hepatic quantitative maps were acquired in a single transversal slice at the level of the bifurcation of portal vein. Relaxation maps were reconstructed directly at the scanner console. Liver MR-elastography was performed with a 2D gradient-recalled echo sequence to acquire liver elasticity maps with motion-encoding gradients. MR-elastography measurements were performed as previously described [8].

### Image analysis

**Fig. 1:**
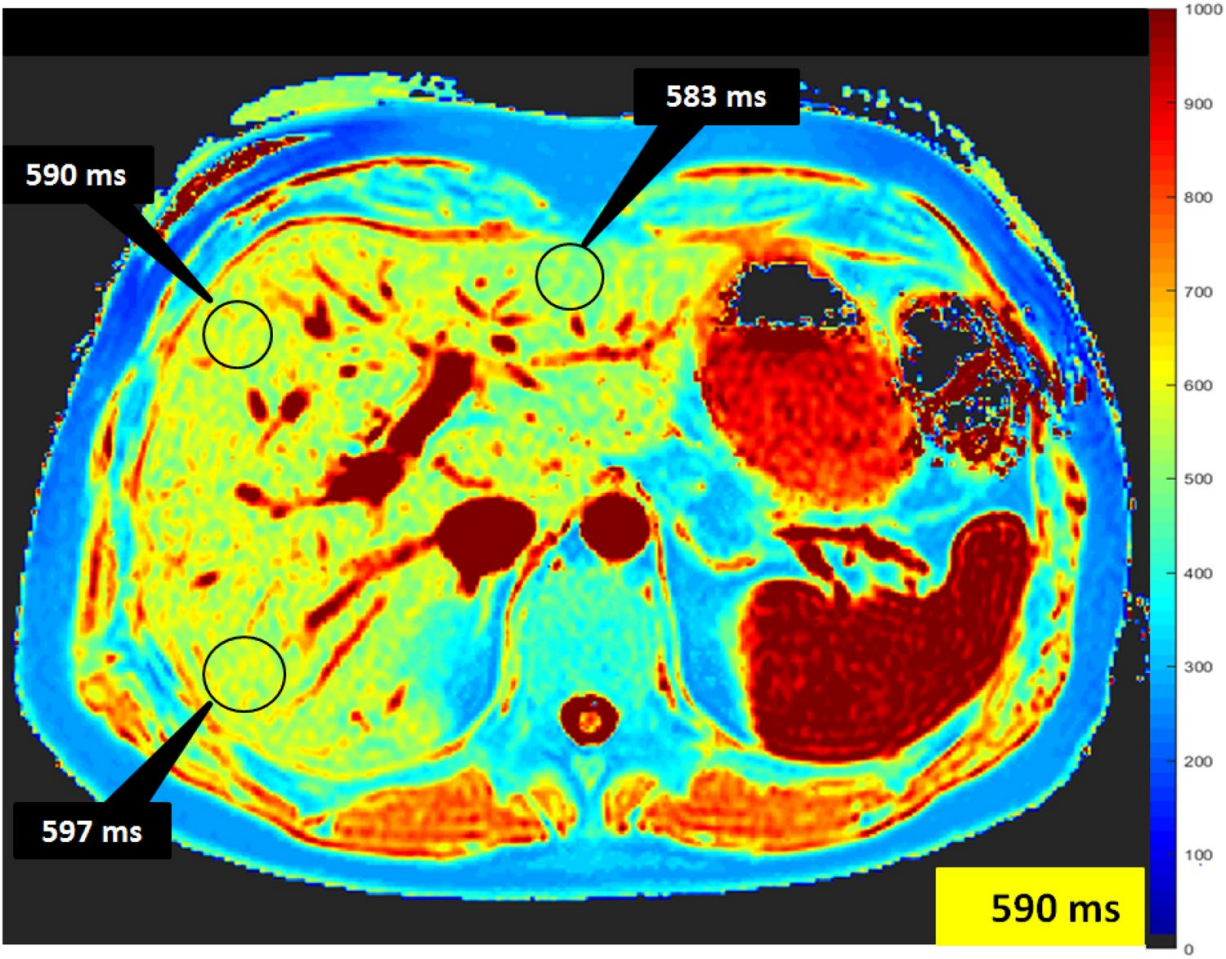
Representative image demonstrating assessment of T1 relaxation times derived from T1 maps. The mean relaxation time of three representative regions of interest drawn centrally in the hepatic segments II, IVa and VII was assessed calculated

An experienced board-certified radiologist (J.A.L, 8 years of experience in abdominal MRI) performed image analyses, blinded to the clinical data. For the assessment of T1 relaxation times, the mean relaxation time of three representative regions of interest (ROI) (≥ 1 cm^2^), drawn centrally in the hepatic segments II, IVa and VII, were calculated (see also Fig. 1). Blood pool T1 values were derived from the abdominal aorta. In the derivation cohort as well as whole study cohort conventional ECV values were normalized for blood Hct and calculated with ROI-based on pre- and post-contrast T1 values according to the previously published equation [29]: ECV = (1 − hematocrit) × (1/T1 parenchyma post-contrast − 1/T1 parenchyma pre-contrast)/(1/T1 aortic post-contrast − 1/T1 aortic pre-contrast).

### Proof-of-concept: synthetic hepatic ECV calculation

The longitudinal relaxivity of blood (R1 = 1/T1) demonstrate a linear relationship with blood Htc, and is determined by the relaxivity of the water fractions of plasma (R1_P_) and the erythrocyte cytoplasm (R1_RBC_) [17]: R1_blood_ = R1_p_ × (1 − Hct) + R1_RBC_ × Hct. Hence, synthetic Hct was derived from the linear relationship between Hct and R1_blood_ and used to calculate synthetic ECV. Synthetic ECV was normalized for synthetic Hct and calculated using the same equation as conventional ECV. Synthetic and conventional ECV were then compared.

### Statistical analysis

Statistical analysis was performed using software (SPSS Statistics, version 25, IBM; Prism 8, GraphPad Software). Patient characteristics are presented as mean ± standard deviation or as absolute frequency, as appropriate. Student t test was used for comparison of continuous variables between two different groups. Dichotomous variables were compared using the *χ*^2^ test (with the cell count > 5) and Fisher test (with a cell count ≤ 5). A locally derived synthetic ECV was created from the longitudinal relaxivity of blood (R1, or 1/T1). This model was created using linear regression, where R1 is the predictor variable and the measured Hct is the outcome. The bivariate Pearson correlation coefficient (*r*) was used for a correlation analysis between synthetic and blood Hct as well as synthetic and conventional ECV. Agreement between individual sets of blood and synthetic Hct as well as conventional and synthetic ECV was analyzed and represented graphically using the Bland–Altman method. The level of statistical significance was set to *P* < 0.05.

## Results

### Cohort characteristics

A total of 180 consecutive patients were included. In the whole study cohort, 87.8% (158/180) of patients had diffuse liver disease and 12.2% (22/180) of patients did not have diffuse liver disease based on past medical history, clinical and laboratory examinations as well as MR-elastography. The mean MR-elastography derived liver stiffness in the group of patients without chronic and/or fibrotic liver disease was 2.1 ± 0.5 kPa. This group of patients consisted of patients with indications for liver MRI examinations as follows: non-specific abdominal symptoms, e.g., non-specific abdominal pain (11/22, 50.0%) or liver lesions detection or/and characterization (11/22, 50.0%). Indications for all MRI examinations in patients with diffuse liver disease were follow-up and/or malignancy exclusion by known chronic liver disease. Etiologies of liver diseases included: alcoholic liver disease (*n* = 27, 15.0%); autoimmune liver diseases, including autoimmune hepatitis, primary sclerosing cholangitis, and primary biliary cirrhosis (*n* = 80, 44.4%); non-alcoholic fatty liver disease (*n* = 12, 6.7%); viral hepatitis (*n* = 13, 7.2%) and other rare etiologies such as portal sinusoidal disease, Budd–Chiari syndrome and Fontan-associated hepatopathy (*n* = 5/180, 2.8%) as well as cryptogenic hepatopathy (*n* = 21, 11.7%). All patients were randomly divided into the derivation (*n* = 90) and validation (*n* = 90) cohorts. The derivation cohort was used to establish the linear regression equation for calculation of synthetic Htc and ECV. The clinical characteristics of the derivation and validation cohorts are presented in Table 1.

**Table 1:** Clinical characteristics of patients in the validation and derivation cohorts

Variable	Derivation cohort (*n* = 90)	Validation cohort (*n* = 90)	*P* value
Age (years)	47.7 ± 16.7	48.6 ± 15.4	0.71
Body mass index (kg/m^2^)	25.6 ± 4.8	25.3 ± 5.3	0.71
Sex			0.88
Male	48	47	
Female	42	43	
Blood hematocrit level (%)	38.5 ± 6.1	39.6 ± 5.1	0.21
Underlying liver disease			
Primary sclerosing cholangitis (PSC)	22 (24.4%)	21 (23.3%)	0.88
Autoimmune hepatitis (AIH)	8 (8.9%)	9 (15%)	0.88
AIH/PSC overlap syndrome	8 (8.9%)	8 (8.9%)	1.00
Primary biliary cirrhosis	2 (22.2%)	2 (22.2%)	1.00
Alcoholic liver disease	14 (15.5%)	13 (7.2%)	0.88
Viral hepatitis	6 (6.7%)	7 (3.9%)	0.88
Non-alcoholic fatty liver disease (NASH)	6 (6.7%)	6 (6.7%)	1.00
Portal sinusoid disease	1 (1.1%)	1 (1.1%)	1.00
Unknown	11 (15.0%)	10 (8.9%)	0.88
Fontan-associated hepatopathy	1 (1.1%)	1 (1.1%)	1.00
Budd–Chiari syndrome	0 (0%)	1 (1.1%)	
No chronic liver disease	11 (12.2%)	11 (12.2%)	1.00
Laboratory parameters			
Bilirubin (mg/dl)	1.33 ± 1.9	1.0 ± 0.74	0.16
ALT (U/l)	77.0 ± 146.1	62.6 ± 87.7	0.43
AST (U/l)	69.7 ± 105.3	51.1 ± 48.5	0.14
GGT (U/l)	182.7 ± 230.5	121.9 ± 150.6	0.04
Platelets cells × 10^9^/l	222.1 ± 109.7	228.1 ± 111.9	0.72
C-reactive protein level (mg/l)	11.6 ± 21.3	5.2 ± 7.7	0.01
AP (U/l)	178.2 ± 181.1	122.4 ± 84.6	0.01
Creatinine (mg/dl)	0.86 ± 0.29	0.90 ± 0.41	0.45
Albumin (g/l)	39.0 ± 10.4	41.2 ± 8.4	0.18
International normalized ratio	1.16 ± 0.36	1.09 ± 0.16	0.11
ASL/ALT (de-Ritis)	1.27 ± 0.9	1.07 ± 0.57	0.07
FIB-4	2.9 ± 3.5	2.3 ± 2.8	0.22
MELD	9.0 ± 4.5	8.43 ± 3.6	0.35
APRI	1.01 ± 1.57	0.76 ± 0.98	0.20

Continuous data are means ± standard deviations. Nominal data are absolute frequencies with percentages in parentheses

*MELD* Score Model of End Stage Liver Disease, *ALT* alanine aminotransferase, *AST* aspartate aminotransferase, *AP* alkaline phosphatase, *GGT* gamma-glutamyltransferase, *APRI* aspartate aminotransferase-to-platelet ratio index, *FIB-4* fibrosis-4 score, *ASL/ALT (de-Ritis)* De-Ritis ratio

### MRI results

#### Derivation cohort

**Fig. 2:**
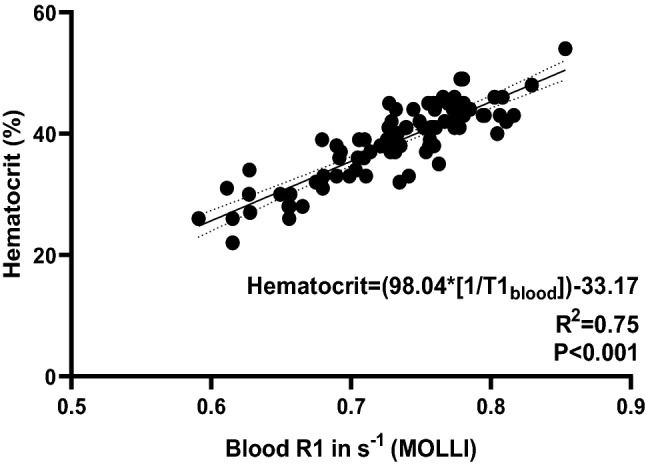
Derivation cohort: Correlation R1_blood_ versus hematocrit using abdominal T1 MOLLI mapping sequence. The regression line between hematocrit and pre-contrast R1_blood_ was linear with *R*^2^ = 0.75, *P* < 0.001 with regression equation as given in the graph. Regression line is given with 95% confidence interval. *MOLLI* modified Look-Locker Inversion Recovery

**Fig. 3:**
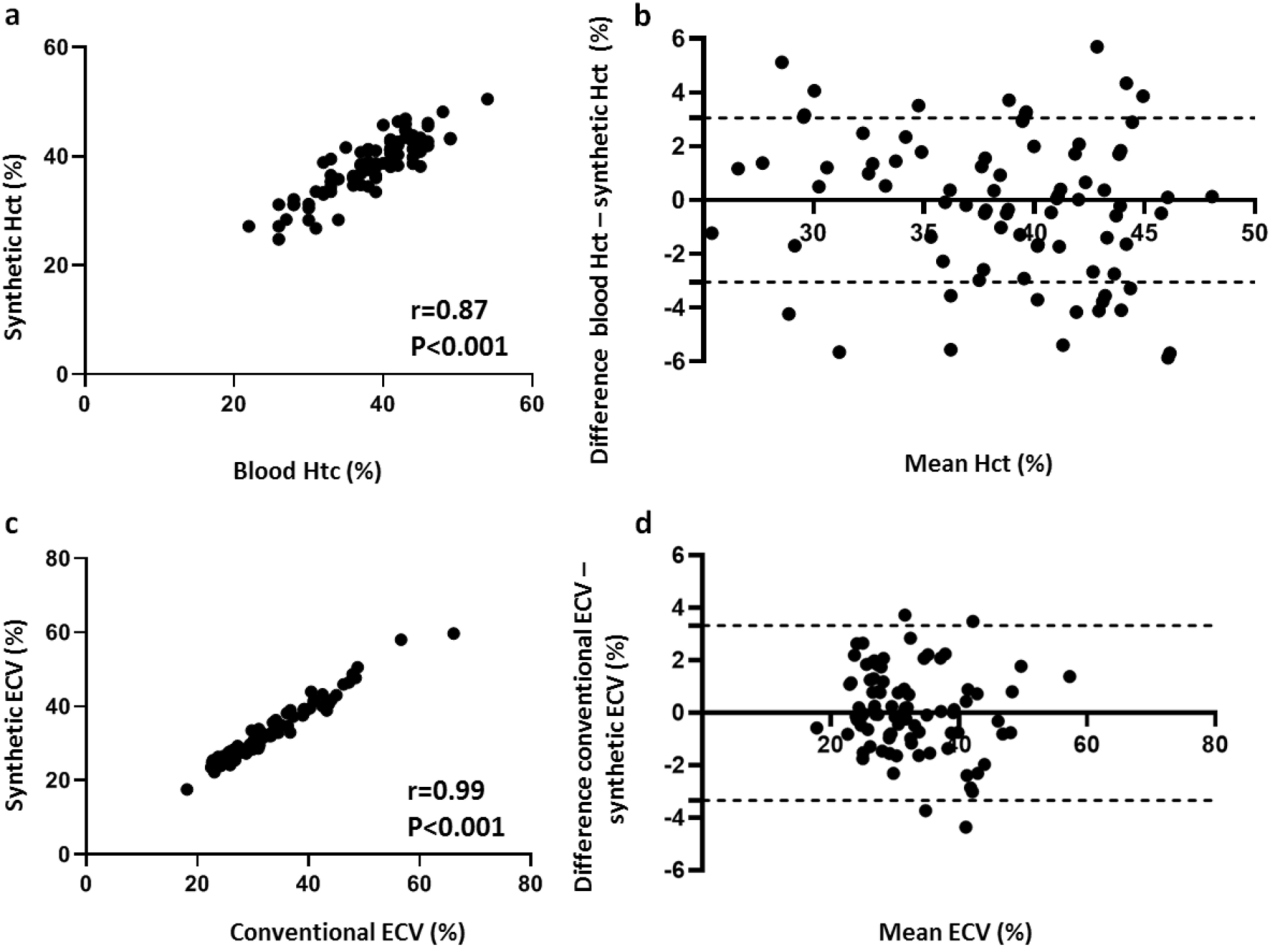
Derivation cohort: synthetic versus blood hematocrit (**a**, **b**) as well as synthetic versus conventional ECV (**c**, **d**). Scatter plots shows correlations between synthetic and blood Hct as well as synthetic and conventional ECV (*n* = 90) (**a**, **c**). Bland–Altman plots of mean differences between blood and synthetic Htc as well as conventional and synthetic ECV. The mean value of measurements for both approaches is plotted on the *x*-axis and the difference between techniques is plotted on the *y*-axis. The solid black horizontal line plots the mean difference and the dotted black lines indicate the limits of agreement (differences from the mean of 1.96 SDs) for each parameter (**b**, **d**). *Htc* hematocrit, *ECV* extracellular volume fraction

For the applied hepatic T1 MOLLI mapping sequence, the regression line between hematocrit and R1_blood_ was linear with *R*^2^ = 0.75, *P* < 0.001. The regression equation for Htc was: Synthetic Hct_MOLLI_ = 98.04 × (1/T1_blood_) − 33.17, where Hct is hematocrit (1 to 100%) and R1_blood_ = 1/T1_blood_ in 10^−3^ s (see also Fig. 2). No significant differences in blood and synthetic Hct (38.5 ± 6.1% vs. 38.5 ± 5.3%, *P* > 0.05) as well as between conventional and synthetic ECV (32.7 ± 8.5% vs. 32.6 ± 7.9%, *P* > 0.05) were found using the above-mentioned equation. Moreover, we found significant correlations between synthetic and blood Htc (*r* = 0.87) as well as synthetic and conventional ECV (*r* = 0.99), in each case *P* < 0.001 (see also Fig. 3).

#### Validation cohort

**Fig. 4:**
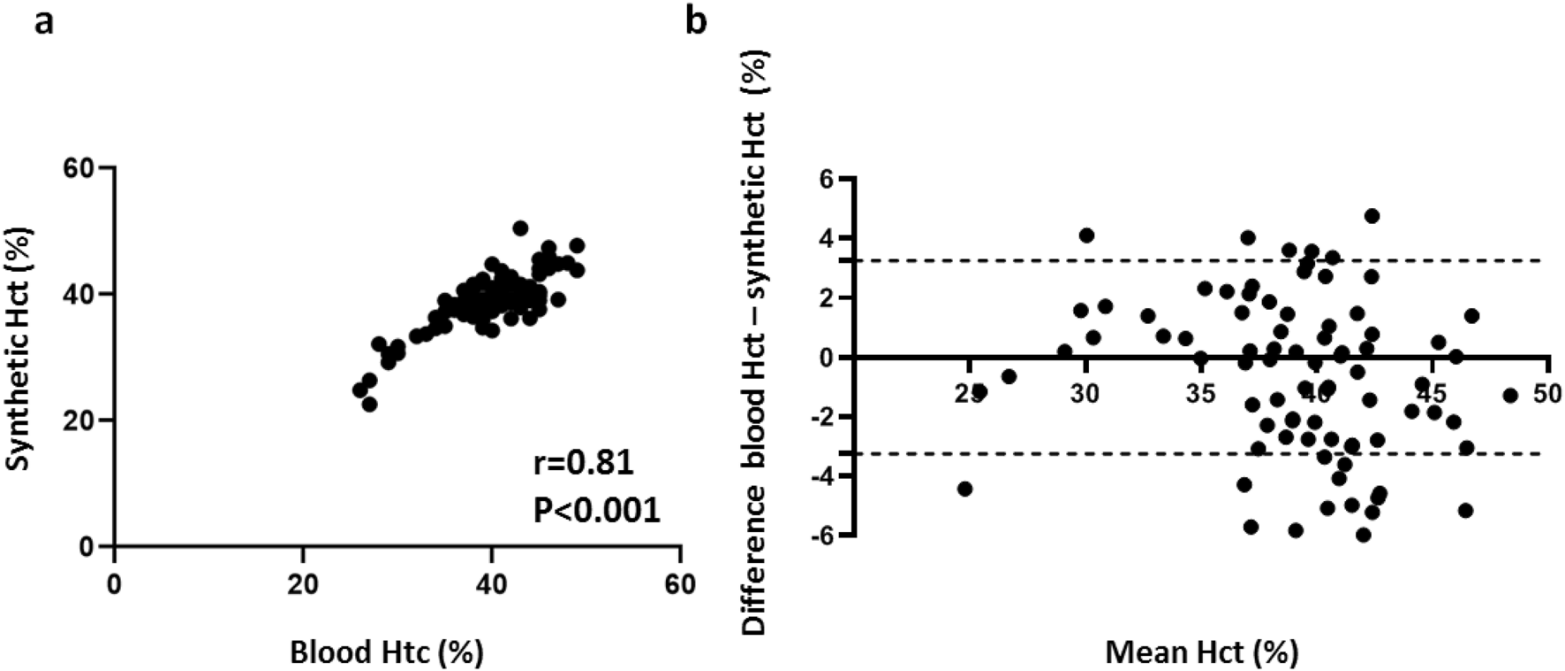
Validation cohort: synthetic versus blood hematocrit. Scatter plots show correlations between synthetic and blood Hct (*n* = 90) (**a**). Bland–Altman plots of mean differences between blood Hct and synthetic Hct. The mean value of measurements for both approaches is plotted on the x-axis and the difference between techniques is plotted on the *y*-axis. The solid black horizontal line plots the mean difference and the dotted black lines indicated the limits of agreement (differences from the mean of 1.96 SDs) for each parameter (**b**). *Htc* hematocrit

**Fig. 5:**
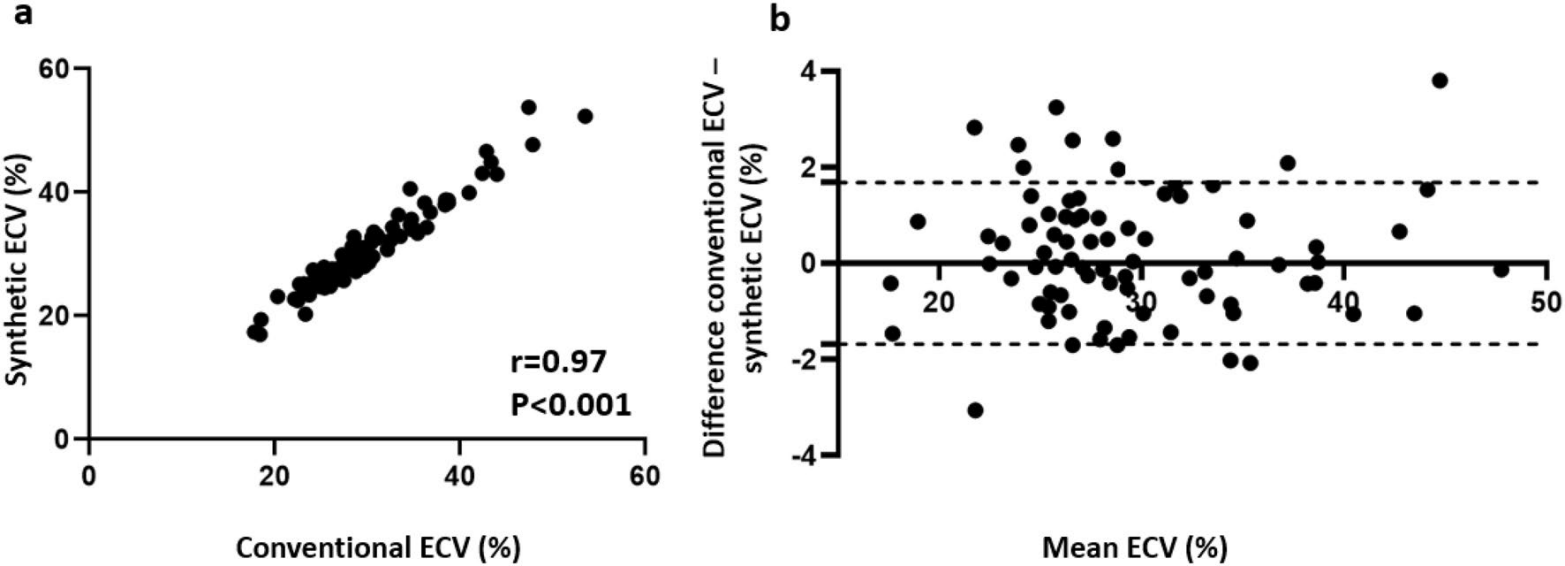
Validation cohort: synthetic versus conventional ECV. Scatter plots show correlations between synthetic and conventional ECV (*n* = 90) (**a**). Bland–Altman plots of mean differences between conventional and synthetic ECV. The mean value of measurements for both approaches is plotted on the *x*-axis and the difference between techniques is plotted on the *y*-axis. The solid black horizontal line plots the mean difference and the dotted black lines indicate the limits of agreement (differences from the mean of 1.96 SDs) for each parameter (**b**). *ECV* extracellular volume fraction

**Fig. 6:**
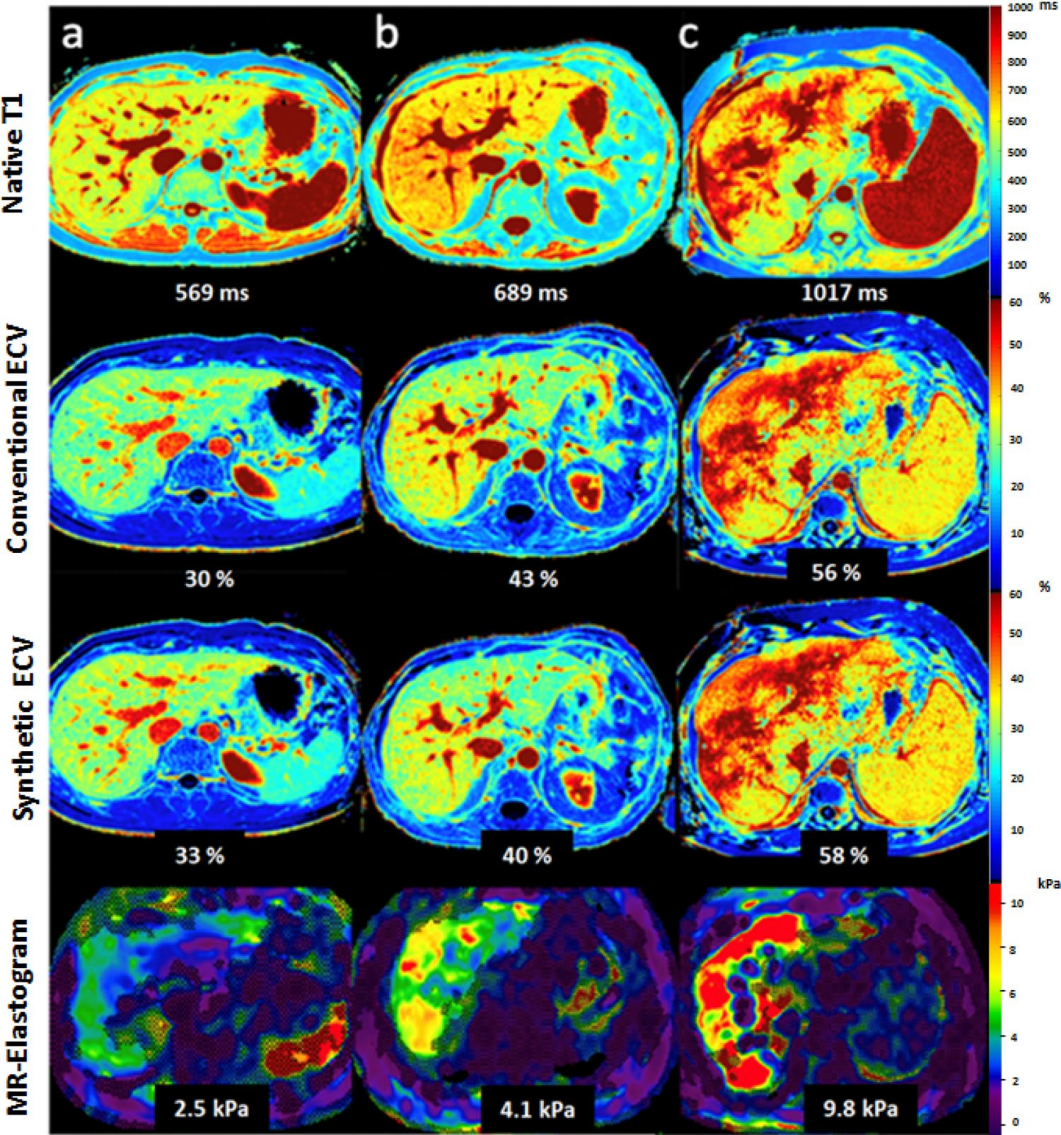
Representative images of conventional and synthetic hepatic extracellular volume (ECV) maps from a 30-year-old male patient with no diffuse liver disease (**a**), from a 24-year-old female patient with autoimmune hepatitis and advanced fibrosis (fibrosis stage (F) 3, **b**) and a 49-year-old male patient with alcoholic liver disease and cirrhosis (F4, **c**) with corresponding MR elastograms. *ECV* extracellular volume fraction

Using above-named equation derived from derivation cohort in the validation cohort, we found no significant differences between blood and synthetic Htc values (39.6 ± 5.1% vs. 38.6 ± 4.8%, *P* > 0.05) as well as conventional and synthetic ECV (30.0 ± 6.7% vs. 30.6 ± 6.9%, *P* > 0.05). Moreover, synthetic and conventional ECV were highly correlated (*r* = 0.97, *P* < 0.001). Synthetic and blood Hct also correlated well (*r* = 0.81, *P* < 0.001). Bland–Altman analysis demonstrated minimal bias for both Hct (− 0.97 ± 3.25%, 95% limits of agreement: − 7.4% to 5.4%) as well as ECV (0.53 ± 1.67, 95% limits of agreement: − 2.75% to − 3.82%) (see also Figs. 4, 5 and 6). MRI characteristics of patients in the derivation and validation cohorts are presented in Table 2.

**Table 2:** MRI characteristics of patients in the derivation and validation cohorts

Variable	Derivation cohort (*n* = 90)	Validation cohort (*n* = 90)	*P* value
Hepatic native T1 relaxation time (ms)	600.5 ± 108.3	571.5 ± 94.0	0.06
Native T1 relaxation time of blood (ms)	1376.6 ± 106.9	1372.3 ± 99.4	0.78
Conventional extracellular volume fraction (%)	32.7 ± 8.5	30.0 ± 6.7	0.02
Synthetic extracellular volume fraction (%)	32.6 ± 7.9	30.6 ± 6.9	0.06
Synthetic hematocrit (%)	38.5 ± 5.3	38.6 ± 4.8	0.83
MR-elastography derived liver stiffness	4.5 ± 1.6	4.2 ± 1.9	0.46

Continuous data are means ± standard deviations

Moreover, we found also strong correlation between conventional and synthetic ECV in patients with chronic liver disease in the whole study cohort with a Pearson’s correlation coefficient of 0.98 (*P* < 0.001).

## Discussion

The purpose of our study was to (1) create locally derived synthetic Hct values from the linear relationship between blood Hct and the longitudinal relaxivity (R1) of the blood and (2) investigate whether synthetic Hct can be used for reliable and valid calculation of synthetic hepatic ECV compared to conventional hepatic ECV. The main findings of our study are that: (1) synthetic Hct derived from linear regression modeling showed a strong correlation with blood Hct and, (2) synthetic ECV showed a strong correlation with conventional ECV and minimal bias according to the Bland–Altman analysis and, therefore, has a potential to be used as a reliable valid biomarker in routine clinical practice alternatively to conventional ECV.

Liver fibrogenesis in patients with chronic liver disease is a consequence of cellular damage and following regeneration processes, leading to increased production of connective tissue with extracellular matrix components. This process leads to the extension of extracellular space and an increased accumulation of extracellular contrast agents, which is reflected by prolonged native T1 relaxation times and increased ECV of the liver. Therefore, with a growing body of evidence, calculation of ECV is considered a new promising potential biomarker for non-invasive assessment of liver fibrosis [7–9]. Therefore, parametric MRI mapping including ECV requires routine clinical use of mapping beyond morphological sequences. However, calculation of ECV requires hematocrit sampling, which may limit the application and availability of these techniques in routine clinical practice. As a result, attempts have been made to eliminate the necessity for blood Htc through estimation of a synthetic Htc in order to calculate an ECV based on the observed linear relationship between Htc and blood R1 (1/T1_blood_). However, the clinical validity of this approach for abdominal applications has not been established yet. A few recent studies in cardiac MRI already demonstrated that synthetic ECV quantification without blood sampling might be a reliable valid tool compared with conventional ECV [22, 23, 30]. However, to our knowledge, there are still no studies showing whether this is also applicable for calculation of hepatic ECV.

In our study we implemented a simple to obtain synthetic ECV measurement using Hct derived from pre-contrast blood T1. The linear relationship between Hct and R1_blood_ has been sufficiently investigated [17, 19–21, 31, 32], and, therefore, we used R1 for curve fitting. We found strong correlations between blood and synthetic Hct with Pearson’s correlation coefficient of 0.81 and 0.83 in the validation as well as the whole study cohort, respectively (*P* < 0.001 in each case). There were also strong correlations between conventional and synthetic ECV in the validation as well as the whole study cohort with *r* = 0.97 and 0.99, respectively (*P* < 0.001 in each case). As far as the results of this study can be compared with the results of previous cardiac studies, these findings support previous data, demonstrating higher correlations between synthetic and conventional ECV compared to synthetic and blood Hct [22, 23]. On the one hand it could be explained by a considerable error in Htc laboratory tests. On the other hand, ECV has other dependencies and additional terms, making it a more stable and robust parameter [33, 34]. Therefore, there could be more inaccuracy as a result of Hct measurements than that as a result of variations in T1 mapping approaches [35, 36]. However, regardless of excellent linear regression fit and in general strong correlations between blood and synthetic Htc as well as conventional and synthetic ECV values, the main disadvantage of synthetic ECV application is that it might lead to considerable errors in individual cases. According to Bland–Altman analysis these variations in individual measurement sets may reach up to 6% between blood and synthetic Hct and up to 4% between conventional and synthetic ECV (see also Figs. 4 and 5). Although the variations between conventional and synthetic ECV were less than 2%, higher variations may have clinical importance for liver fibrosis staging. Patients could be misclassified in a wrong fibrosis stage, which is especially vital for the detection of significant fibrosis. The presence of even greater variabilities was also demonstrated in previous cardiac studies, with more pronounced differences in Htc than in ECV values [22, 23]. The variability in laboratory Htc and calibration of conventional ECV to blood Hct may also lead to miscategorization. Hence, precise clinical evaluation based on medical history, laboratory examinations as well as MRI (including, e.g., MR-elastography) in individual patients are needed to minimize the possible discrepancies between synthetic and conventional values and therefore its influence on clinical decision-making (Table 3).

**Table 3:** Correlation values for synthetic hematocrit and ECV in the derivation, validation and the whole study cohorts

Variable	Derivation cohort		Validation cohort		Whole study cohort	
	*R* value	*P* value	*R* value	*P* value	*R* value	*P* value
Blood hematocrit vs. synthetic hematocrit	0.87	< 0.001	0.81	< 0.001	0.83	< 0.001
Conventional ECV vs. synthetic ECV	0.99	< 0.001	0.97	< 0.001	0.99	< 0.001

*ECV* extracellular volume fraction

There are several limitations in our study. The main limitation was that the sample size was modest and all examinations were performed in a single center. Furthermore, the fact that T1 mapping techniques vary across the institutions can additionally limit the applicability of our study results. Furthermore, synthetic Hct requires local calibration, unless MRI scanner, used T1 mapping parameters, and machine for Hct laboratory are the same. Moreover, as the accuracy of current T1 measurements method remains to be established, this study does not claim to report an accurate measure of T1, but that synthetic calculation of hepatic Hct derived from used T1 MOLLI sequence is a stable and reliable approach for routine clinical practice. Another significant limitation for clinical application of synthetic measurements is that equations for synthetic Hct calculation should be derived individually on each MRI scanner using the same acquisition scheme. Therefore, if synthetic ECV is to be used in routine clinical practice where blood Hct cannot be obtained, using a locally derived synthetic Hct regression model for the used T1 mapping sequence is preferred.

In conclusion, this is the first study investigating the applicability of synthetic hepatic Hct derived from a regression model for ECV calculation without Htc sampling. Our findings suggest that ECV calculated from synthetic Hct may be a useful, valid and reliable tool compared with conventional ECV. Further multi-centric prospective studies on a larger population are needed to validate these findings across the centers, using different T1 mapping sequences to enable the further clinical implementation of ECV by liver examinations. The use of synthetic ECV may potentially overcome an important barrier for clinical implementation of hepatic ECV measurements.

## Data Availability

The datasets generated and/or analyzed during the current study are available from the corresponding author on reasonable request.

## Abbreviations

MRI: Magnetic resonance imaging
ECV: Extracellular volume fraction
Htc: Hematocrit
